# A single-arm, phase 2 study of adjuvant chemotherapy with oral tegafur-uracil for pathologically lymphovascular invasion positive stage IA non-small cell lung cancer: LOGIK0602 study

**DOI:** 10.1186/s12885-020-07691-7

**Published:** 2020-12-04

**Authors:** Tomoshi Tsuchiya, Ryotaro Kamohara, Masashi Muraoka, Takeshi Nagayasu, Sho Saeki, Mitsuhiro Takenoyama, Makoto Suzuki, Kazuo Inada, Shoji Tokunaga, Tomayoshi Hayashi, Shogo Urabe, Takaomi Koga, Shinji Akamine, Kenji Sugio

**Affiliations:** 1 Department of Surgical Oncology, Nagasaki University Graduate School of Biomedical Sciences, 1-7-1 Sakamoto, Nagasaki City, 852-8501 Japan; 2 Department of Thoracic Surgery, Oita Prefectural Hospital, Oita, Japan; 3 Department of Thoracic Surgery, Japan Community Health care Organization Isahaya General Hospital, Isahaya, Japan; 4 Department of Respiratory Medicine, Kumamoto University Hospital, Kumamoto, Japan; 5 Department of Respiratory Oncology, National Hospital Organization Kyushu Cancer Center, Fukuoka, Japan; 6 Department of Thoracic Surgery, Kumamoto University Hospital, Kumamoto, Japan; 7 Department of Thoracic Surgery, National Hospital Organization Omuta Hospital, Omuta, Japan; 8 Medical Information Center, Kyushu University Hospital, Fukuoka, Japan; 9 Department of Pathology, Nagasaki Prefecture Shimabara Hospital, Nagasaki, Japan; 10 Department of Pathology, Oita Prefectural Hospital, Oita, Japan; 11 Department of Pathology, Pathophysiological and Experimental Pathology, Fukuoka, Japan; 12 Department of Thoracic and Breast Surgery, Oita University, Oita, Japan

**Keywords:** Non-small cell lung cancer, Adjuvant chemotherapy, Tegafur-uracil, Lymphovascular invasion, Targeted therapy, Phase II study

## Abstract

**Background:**

Lymphovascular invasion (LVI), which includes vascular or lymphatic invasions, is a representative prognostic factor even in patients with resected stage IA non-small cell lung cancer (NSCLC). Because tegafur-uracil is effective on cancers with LVI, we conducted a multi-center single-arm phase II study to estimate the efficacy of adjuvant tegafur-uracil in patients with LVI-positive stage IA NSCLC.

**Methods:**

Patients with completely resected LVI-positive stage IA NSCLC were registered. LVI was diagnosed by consensus of two of three pathologists. Adjuvant chemotherapy consisted of 2 years of oral tegafur-uracil at 250 mg/m^2^/day. Fifty-five patients from 7 institutions were enrolled from June 2007 to September 2012.

**Results:**

Among the 52 eligible patients, 36 (69.2%) completed the treatment course. There were 39 male and 13 female patients. The observation period was calculated as 562 to 3107 days using the reverse Kaplan-Meier method. The 5-year overall and relapse free survival rates were 94.2 and 88.5% respectively, which were significantly better than that of any other studies conducted on patients with LVI-positive stage IA NSCLC. Notably, the overall survival rate was 15% better than that of our prior retrospective study. The retrospective analysis of stage IA NSCLC patients who had received an operation in the same period revealed that the 5-year overall survival rate of the LVI positive group was 73.6% when adjuvant chemotherapy was not applied. Among 55 safety analysis sets, 4 cases of grade 3 hepatic function disorder (9.1%) and 5 cases of grade 2 anorexia (10.9%) were most frequently observed. No grade 4 adverse effects were encountered.

**Conclusion:**

A 2-year course of oral tegafur-uracil administration is feasible and might have a significant benefit in the adjuvant treatment of LVI-positive stage IA NSCLC.

**Trial registration:**

UMIN identifier: UMIN000005921; Date of enrolment of the first participant to the trial: 19 June 2007; Date of registration: 5 July 2011 (retrospectively registered).

## Background

Lung cancer remains the leading cause of cancer-related deaths worldwide [1]. A recent public health study indicates an increase in stage IA non-small cell lung cancer (NSCLC) and an improved prognosis for the disease [2]. In NCCN clinical practice guidelines, adjuvant chemotherapy is not recommended for surgical margins negative (R0) stage IA NSCLC. However, stage IA NSCLCs have been sub-divided according to poor prognostic factors such as smoking history, serum level of carcinoembryonic antigen (CEA), resection area, tumor size, lymphatic vessel invasion and vessel invasion [3–12]. Therefore, poor prognostic group of stage IA NSCLC might obtain benefit form adjuvant chemotherapy.

Lymphovascular Invasion (LVI), which includes lymphatic and blood vessel invasion, is thought to reflect tumor aggressiveness and to play a crucial role in the first step of tumor metastasis in a variety of human cancers [13]. Therefore, LVI has been shown to be a strong predictor of unfavourable prognosis even in the curatively resected pathological stage IA NSCLC. Our prospective analysis of 791 postoperative stage I NSCLC patients in two institutions revealed that the overall 5-year survival rate of the stage IA LVI group nearly overlapped with that of patients with stage IB NSCLC [10]. In addition, detailed analysis of 221 stage IA NSCLC patients showed that both lymphatic vessel invasion and blood vessel invasion were significant poor prognostic factors [14]. The meta-analysis of stage I NSCLC of the lung demonstrated that LVI was a strong prognostic indicator even in stage IA disease [15]. Accordingly, clinicians have proposed that patients with LVI-positive stage IA NSCLC may be good candidates for adjuvant chemotherapy [6, 9, 11, 12, 15–20]. However, the targeted adjuvant therapy for LVI-positive stage IA NSCLC has not been conducted so far.

Tegafur-uracil is a first generation dihydropyrimidine dehydrogenase (DPD) inhibitory fluoropyrimidine drug. It is an oral agent which combines uracil, a competitive inhibitor of DPD, with the 5-fluorouracil (5-FU) prodrug tegafur in a 4:1 M ratio. Because DPD degrades 5-FU in the liver and in a variety of cancer cells, tegafur-uracil DPD inhibition enhances the effect of 5-FU metabolized from tegafur. A phase III clinical study showed that treatment with tegafur-uracil improved survival rates of patients with stage IB and with an adenocarcinoma tumor measuring 2 to 3 cm in diameter [21]. Interestingly, in a mouse model, tegafur-uracil and the metabolites of 5-FU and γ-hydroxybutyric acid significantly inhibit vascular endothelial growth factor (VEGF) mediated angiogenesis induced by many human cancer cell lines [21, 22] and VEGF mediated angiogenesis correlates with tumor vessel invasion in NSCLC [23]. Therefore, we speculated that tegafur-uracil could kill circulating cancer cells generated from resected LVI positive tumors, which would improve the survival of patients with LVI-positive stage IA NSCLC using adjuvant treatment. In fact, our previous retrospective analyses of 322 cases of stage IA NSCLC identified that when administered, the tegafur-uracil adjuvant chemotherapy increased the overall 5-year survival rate of the stage IA LVI-positive group by more than 25% (93.3% for the treatment group and 66.6% for the no-treatment group; *P* = 0.036) [9].

According to the theoretical assumption and previous analysis, we conducted a multicenter single arm phase II study of adjuvant chemotherapy with tegafur-uracil for poor prognostic patients with pathologically LVI positive stage IA NSCLC (LOGIK0602). The objective of the present study is to improve the prognosis of patients with stage IA LVI-positive NSCLC using adjuvant chemotherapy of oral tegafur-uracil.

## Methods

### Patient eligibility

The study was conducted in the Lung Cancer Group in Kyusyu (LOGIK). Eligible patients were enrolled from September 2007 to April 2010. Patient eligibility required compliance with the following criteria: NSCLC with histological proof; surgically resected pathological stage IA NSCLC (according to the 7th edition of UICC/AJCC, 2010) [24]; no prior treatment; age > 45 and < 80 years, with sufficient oral intake; and performance status (PS) 0 or 1. Patients also needed adequate organ function: (leukocytes ≤4000/≤12,000/mm^3^; thrombocytes ≥100,000/mm^3^; total bilirubin ≤1.5 mg/dL; aspartate aminotransferase (AST) and alanine aminotransferase (ALT) less than twice the normal limits at each site; blood urea nitrogen ≤25 mg/dL; creatinine less than the normal limits at each site. Patients with a history of drug hypersensitivity, serious surgical or non-surgical complications, or active secondary cancer were excluded. Pregnant or lactating women were likewise excluded.

### Pathological diagnosis for LVI

LVI was defined as the presence of neoplastic cells within an arterial, venous, or lymphatic lumen. According to the stomach cancer and colon cancer handling conventions, LVI was judged by HE staining and EVG staining as previously described [9, 10, 14]. The analysed slides were made by maximum cut-surface of NSCLC and sent to three pathologists in three institutions in turn. The diagnosis of LVI was made by consensus of two of three pathologists’ diagnosis.

### Criteria for oral tegafur-uracil treatment

The criteria of the oral tegafur-uracil treatment were determined as follow. Tegafur-uracil (250 mg of tegafur per square meter of body surface area per day) in the form of 100-mg capsules (100 mg of tegafur plus 224 mg of uracil) was administered orally before meals twice daily for 2 years, starting within 4 to 8 weeks after surgical resection of the lung tumor. Most patients received two capsules of tegafur-uracil (200 mg of tegafur and 448 mg of uracil) twice daily.

### Evaluation of the response and toxicities

The Common Terminology Criteria for Adverse Events, Version 4.0 (CTCAE, 2009) were adopted for the evaluation of chemotherapy toxicity.

### Study design and statistical analysis

This trial was non-blinded and open label. The primary end point was 5-year overall survival rate of treated patients. Secondary end points were the completion rate of the scheduled adjuvant chemotherapy, grade of adverse reactions, relapse rate, and 5-year relapse free survival rate.

With null hypothesis of 70% of 5-year survival rate and an increase in 5-year survival rate by 15% point, the minimum number of patients needed to attain statistical power over 80% assuming one sample binomial test with one-sided alpha of 5% was 49. Considering around 10% drop outs, the number of patients to be enrolled was set to 55. The Kaplan-Meier method was used to estimate the time-to-event functions of relapse-free survival and overall survival. Relapse-free survival has been defined as the time from the date of the start of treatment to the date of disease progression or death (whichever occurs first) or the date of last contact. Overall survival has been defined as the time from the date of the start of treatment to the date of death or last contact. The log-rank test was used to test for possible differences between estimated time-to-event curves.

### Survival analysis of retrospective prognosis data

The patient prognosis data was retrieved from Nagasaki University lung cancer database. The retrieved patient data was selected by the following eligibility criteria: surgically resected pathological stage IA NSCLC with histological proof (according to the 7th edition of UICC/AJCC, 2010) [24] with no prior treatment or adjuvant chemotherapy; age > 45 and < 80 years. The Kaplan-Meier method was used to estimate the time-to-event functions of overall survival from the surgery. The log-rank test was used to test for possible differences between estimated time-to-event curves.

## Results

### Patient characteristics

A total of 55 patients were initially enrolled in the present study. Safety analysis set (SAS) was 55 because all patients were treated by tegafur-uracil postoperatively. Three patients were deemed ineligible because two patients had an earlier start of therapy before registration and one patient had a history of cancer treatment within 5 years before registration. Thus, full analysis set (FAS) was 52. Table 1 shows the characteristics of the 52 eligible patients. There were 39 male and 13 female patients. The observation period was 562–3107 days. The median follow-up period was 1947 days. The mean age of the patients was 67.5 years (range, 47–78 years). All patients showed a performance status of 0. Of the 52 eligible patients, 5 were 1 cm or less at its widest solid part (9.6%), 30 were between 1 cm and 2 cm across (57.7%), and 17 were between 2 cm and 3 cm across (32.7%).

**Table 1:** Patient Characteristics (*n* = 52)

Variables	n	Percentage
Sex		
Male	39	75
Female	13	25
Age (years)	Mean, 67.5 (47 ~ 78)	
Tumor size		
1 cm or less	2	3.8
1 cm <, 2 cm or less	26	52.0
2 cm <, 3 cm or less	24	46.2
Solid part size		
1 cm or less	5	9.6
1 cm <, 2 cm or less	30	57.7
2 cm <, 3 cm or less	17	32.7
CT image		
Solid (CRR = 1.0)	33	63.5
Sub-solid (0.5≦CTR < 1.0)	17	32.7
Mixed GGN (CTR < 0.5)	2	3.8

*CTR* Consolidation tumor ratio, *GGN* Ground grass nodule

### Drug compliance

Among the 52 eligible patients, 36 (69.2%) completed the planned 2-year course of tegafur-uracil treatment and 16 (30.8%) discontinued the administration. Among the 36 treatment-completed patients, 4 patients received dose reduction (11.1% of 36 patients). Among 16 treatment-discontinued patients, 11 patients (68.8% of 16 patients) stopped tegafur-uracil administration without dose reduction.

Table 2 shows drug compliance in the 2-year course and reasons for discontinuation of drug administration. Main iatrogenic reasons were anorexia, malaise, diarrhoea, elevation of AST or ALT, and elevation of total bilirubin. Non-iatrogenic reasons for discontinuation were seven cases of patient refusal with or without low grade (1 or 2) adverse reactions (43.8% of 16 patients). In addition, severe adverse reactions occurred after a brief interval from the start of the treatment.

**Table 2:** Drug Compliance (N = 52)

Discontinuation Day	No. of Patients	Completion Rate (%)	Reason for Discontinuation
	52	100	
5	51	98.1	Grade 1 anorexia (Patient refusal)
5	50	96.2	Grade 2 malaise (Patient refusal)
42	49	94.2	Recurrence
47	48	92.3	Grade 1 diarrhea (Patient refusal)
134	47	90.4	Grade 3 elevation of AST or ALT
141	46	88.5	Grade 3 elevation of total bilirubin
171	45	86.5	Patient refusal
217	44	84.6	Patient refusal
245	43	82.7	Grade 3 body weight loss
267	42	80.8	Grade 2 elevation of AST or ALT
311	41	78.8	Grade 3 malaise
321	40	76.9	Grade 2 dry skin
403	39	75.0	Grade 2 nausea (Patient refusal)
455	38	73.1	Grade 2 pain of skin (Herpes zoster)
505	37	71.2	Grade 2 interstitial pneumonia
682	36	69.2	Patient refusal

### Adverse reactions

Table 3 shows a summary of the adverse reactions encountered. The data of 55 patients in SAS were used for the analysis. Among the laboratory findings-based adverse reactions, elevation of AST or ALT was the most frequent, occurring in 5 of the 52 patients (9.1%), followed by neutropenia (1.8%), elevation of creatinine (1.8%), and hyper bilirubinemia (1.8%). Among the clinical findings based adverse reactions, anorexia was the most frequent (10.9%), followed by nausea (9.1%), and diarrhoea (7.3%), malaise (3.6%), skin disorder (3.6%), pneumonia (1.8%), and weight loss (1.8%). Concerning the incidence and grade of laboratory findings-based adverse reactions, grade 3 adverse reactions were seen with elevation of AST or ALT, hyper bilirubinemia, anorexia, nausea, and malaise. No grade 4 adverse reactions were identified.

**Table 3:** Adverse reactions

Grades of Adverse Reactions	G1	G2	G3	G4	Total (Incidence %)
Neutropenia	0	1	0	0	1.8
Anemia	0	0	0	0	0
Elevation of AST or ALT	0	1	4	0	9.1
Elevation of creatinine	1	0	0	0	1.8
Hyper bilirubinemia	0	0	1	0	1.8
Anorexia	0	5	1	0	10.9
Nausea	2	2	1	0	9.1
Diarrhea	2	2	0	0	7.3
Malaise	0	1	1	0	3.6
Skin disorder	0	2	0	0	3.6
Pneumonia	0	1	0	0	1.8
Weight loss	0	0	1	0	1.8
Others	1	1	0	0	3.6

### Survival

**Fig. 1:**
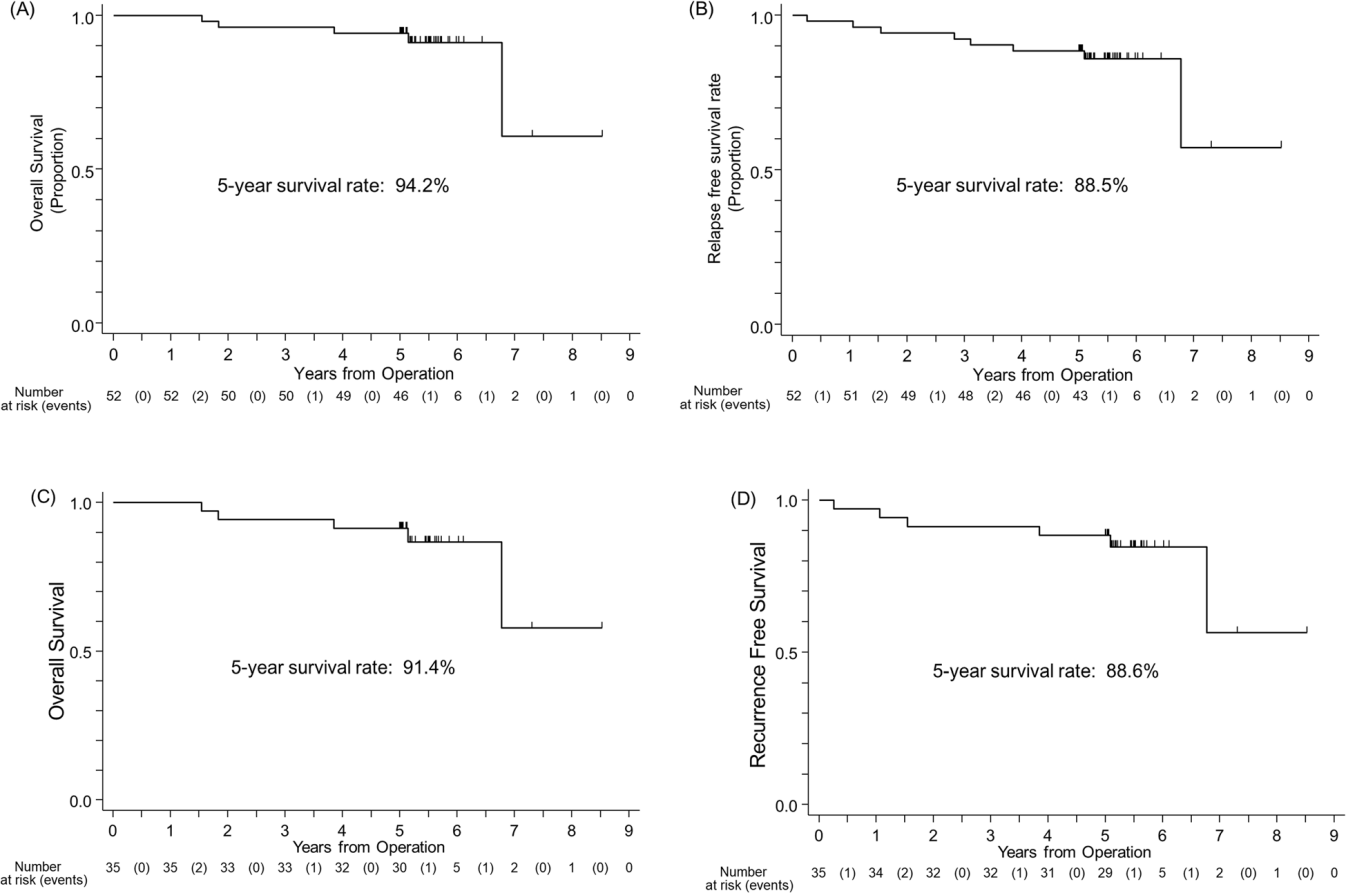
Kaplan-Meier analysis of (**a**) overall and (**b**) relapse-free survival of patients who were treated with tegafur-uracil for LVI-positive stage IA NSCLC in adjuvant setting. Kaplan-Meier analysis of (c) overall and (d) relapse-free survival of patients who were treated with tegafur-uracil for 2 cm or less size LVI-positive stage IA NSCLC in adjuvant setting. LVI, lymphovascular invasion; NSCLC, non-small cell carcinoma

Among the 52 patients followed for survival information, only 3 had died and 49 were alive at the time of 5-yr analysis. The observation period was calculated as 562 to 3107 days and median observation period as 1947 days. The 3-yr and 5-yr overall survival rates were 96.2% (95% CI, 85.5–99.0) and 94.2% (95% CI, 83.2–98.1), respectively (Fig. 1a). Of the 3 patients who died prior to 5-yrs, one had experienced a documented relapse before death. Two patients died of pneumonia and respiratory failure. A total of 5 patients relapsed at the time of 5-yr analysis, and the 3-yr- and 5-yr- relapse free survival rates were 92.3% (95% CI, 80.8–97.0) and 88.5% (95% CI, 76.1–94.6), respectively (Fig. 1b). The analysis of smaller sized 2 cm or less tumor showed that the 5-yr overall and relapse free survival rates were 91.4 and 88.6%, respectively (Fig. 1c and d).

The overall and relapse-free survival rates for patients with NSCLC in this study were significantly higher than that of any other studies conducted on the postoperative patients with lymphatic or vessel invasion-positive stage IA NSCLC. The overall survival rate was 15% better than the data from our previous retrospective study (Table 4) [9]. Among the patients who experienced relapse, 4 patients experienced intrathoracic recurrence, including 1 with lung metastasis, 1 with regional lymphatic metastasis with dissemination, and 1 with regional lymphatic metastasis. One patient showed distant metastasis at the adrenal gland. One patient suffered a newly developed lung cancer at the opposite side of the upper lobe of the lung.

**Table 4:** Historical Survival Data of the Patients with BVI, LyVI or LVI Positive Stage IA NSCLC

Author	Year	T-Classification	Vessel invasion status	Number of Patients	5-year Survival Rates (%)	
					Overall	Relapse free
Pechet et al. [6]	2004	T1	BVI	22	28	–
Tsuchiya et al. [9]	2007	T1	LVI (BVI or LyVI)	85	71.8	–
Tsuchiya et al. [10]	2007	T1	LVI (BVI or LyVI)	144	78.7	–
Miyoshi et al. [11]	2009	T1	LVI (BVI or LyVI)	62	78	–
Funai et al. [12]	2011	T1	LyVI	22	70.9	–
Ito et al. [16]	2012	T1	LVI (BVI or LyVI)	105	–	58
Shimada et al. [14]	2012	T1	BVI	116	72.1	71.3
			LyVI	122	76.4	76.1
Kudo et al. [17]	2013	T1a	BVI	38	87.1	72.5
		T1b	BVI	39	65.9	58.9
Hamanaka et al. [19]	2015	T1	LVI (BVI or LVI)	56	–	79.7
**Present series**	**2018**	**T1**	**LVI (BVI or LVI)**	**52**	**94.2**	**88.5**

*BVI* Blood vessel invasion, *LVI* Lymphovascular invasion, *LyVI* Lymphatic vessel invasion

### Analysis of the retrospective data of stage IA NSCLC

**Fig. 2:**
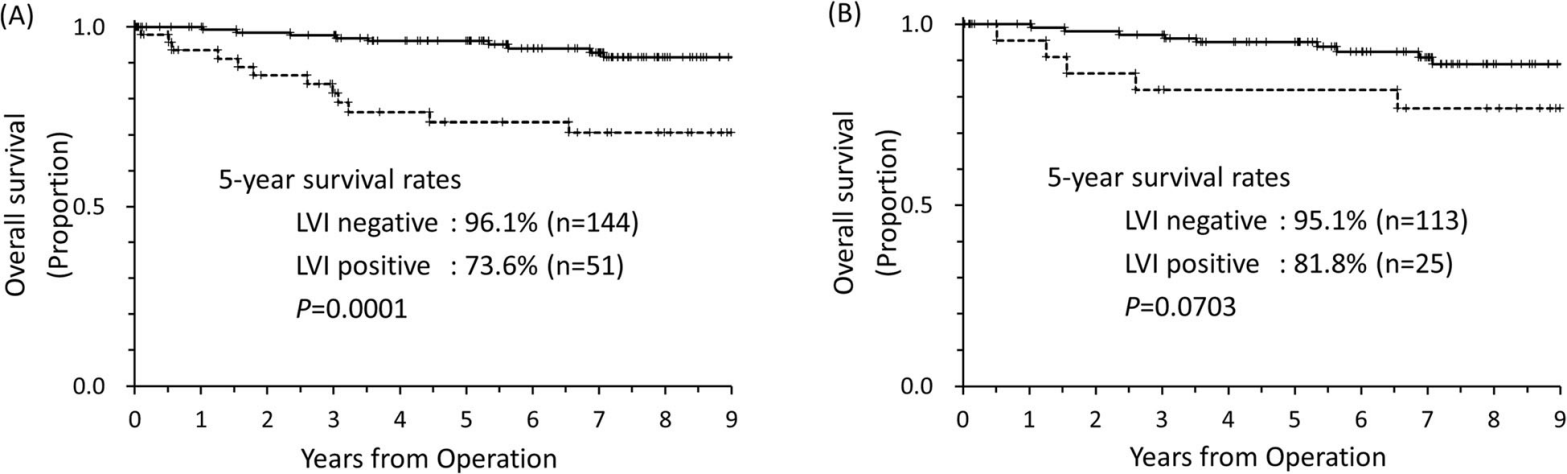
5-year overall survival rates of retrospectively analysed postoperative patients without adjuvant chemotherapy. **a** Stage IA NSCLC with or without LVI. **b** 2 cm or less size Stage IA NSCLC with or without LVI. LVI, lymphovascular invasion; NSCLC, non-small cell carcinoma

In order to estimate the benefit of tegafur-uracil treatment of the LVI-positive stage IA NSCLC patients, the survival data of the postoperative patients who had not received adjuvant chemotherapy was analysed by log-rank test. During the same period of registration for the LOGIK0602 study, 195 patients qualified under the criteria. The 5-yr overall survival rate of LVI-negative patients was 96.1 and 73.6% for patients positive for LVI, respectively (*p* = 0.0001) (Fig. 2a). The analysis of smaller sized 2 cm or less NSCLC showed that the 5-yr overall survival rate of LVI-negative patients was 95.1% and LVI-positive patients was 81.8%, respectively (*p* = 0.0703) (Fig. 2b).

## Discussion

The present study identified significant benefits of tegafur-uracil for the LVI-positive stage IA NSCLC. The 94.2% of overall survival rates in this study were more than 15% higher than survival rates from our previous study (Table 4) [9]. In addition, the retrospective analysis of postoperative stage IA NSCLC patients showed that the 5-year survival rate of the LVI-positive group was 20% lower than the LOGIK0602 study, suggesting that tegafur-uracil based adjuvant chemotherapy definitely improves the prognosis of LVI-positive stage IA patients. To our knowledge, this is the first report which indicates the benefit of targeted adjuvant chemotherapy for a poor prognostic group of stage IA NSCLC. Although tegafur-uracil is not well known in Western countries, the seminal prospective study of Kato et al. indicated that patients with a tumor measuring 3 cm or more in diameter obtained significant benefit from tegafur-uracil treatment [21]. Therefore, tegafur-uracil adjuvant chemotherapy has been performed for surgically treated patients with stage IB lung cancer in Japan. In addition, a randomized controlled study to compare the survival benefit of paclitaxel plus carboplatin and tegafur-uracil treatment as adjuvant chemotherapy showed that, paclitaxel plus carboplatin was no better than tegafur-uracil in terms of survival among patients with stage IB to IIIA NSCLC [25]. Our results and the data indicate that tegafur-uracil treatment might have sufficient power to improve survival in a poor prognosis group of postoperative patients with stage IA NSCLC.

The present study was also undertaken to confirm the feasibility of 2-year oral adjuvant chemotherapy with tegafur-uracil after complete resection for stage IA NSCLC. The completion rate for the planned 2 years of tegafur-uracil administration was 69.2%, which compares favorably with the chemotherapy compliance seen in trials of Kato and colleagues (80%) and cisplatin plus vinorelbine based adjuvant therapies (48 and 50%) [21, 26, 27]. On the other hand, a detailed analysis of sixteen discontinued cases identified that seven cases were discontinued by patient refusal or upon their doctors’ judgement without reaching discontinuation criteria (43.8% of 16 patients). In addition, 11 patients (68.8% of 16 patients) stopped administration without dose reduction. Because the duration of the therapy was for 2-years, patients might find going to the hospital for the treatment problematic and might discontinue therapy during the middle or end of the treatment course.

Toxicity in the present study was significantly less compared with the cisplatin-based adjuvant therapies. The studies of cisplatin plus vinorelbine based postoperative adjuvant chemotherapy have indicated that the frequency of grade 3/4 adverse reactions was more than 49% [26, 27]. Conversely, throughout the 2-year tegafur-uracil administration, no grade 4 adverse reactions were observed and only five (9.1%) hematological and four (7.3%) non-hematological G3 adverse reactions were identified. The administration of tegafur-uracil for outpatients was easily continued because the most common adverse reaction was grade 2 anorexia in 9.1% of patients. Accordingly, tegafur-uracil is considered to be an attractive oral anticancer agent for postoperative adjuvant chemotherapy with very low toxicity.

Tegafur-uracil inhibits cancer-induced angiogenesis mediated by the vascular endothelial growth factor (VEGF) related pathway [22]. Interestingly, VEGF expression significantly correlates with LVI in lung cancer [23] and oral tegafur-uracil treatment significantly increased the survival rate of pathologic stage I NSCLC patients with VEGF overexpressing tumors [28]. Therefore, it is possible that NSCLC with LVI has a high sensitivity for tegafur-uracil treatment and patients with LVI positive stage IA NSCLC would benefit from such treatment. Another advantage of tegafur-uracil usage in adjuvant setting is that cisplatin based chemotherapy can be preferentially applied after recurrence because tegafur-uracil belongs to the antimetabolite and has a different anticancer mechanism from cisplatin, The advantage of tegafur-uracil treatment is that it expands treatment options and prolongs the survival of relapsed patients. This might explain the discrepancy of 5-yr overall survival of 94.2% and 5-yr relapse free survival of 88.5% in the present study.

One difficulty of the present study was distinguishing among capillary size of arterial, venous, or lymphatic lumens. Pathologists usually differentiate blood vessels from lymphatic vessels according to the evidence of blood cells in endothelium-lined channels. Elastic stains are also used in some institutions as a routine examination for blood vessel invasion because lymphatic vessels do not contain elastic fibers. However, there is a possibility that the diagnostic accuracy of lymphatic vessel invasion and blood vessel invasion differ among pathologists [10]. In addition, the lymphatic invasion and blood vessel invasion often co-existed and the occurrences were correlated [14]. Therefore, we set aside the separate estimation of lymph or blood vessel invasion and focused on the increase of the accuracy of the LVI diagnosis. Accordingly, we applied the three pathologists’ agreement using H&E staining and EVG staining. Although it can be done on routinely fixed paraffin-embedded tissues nowadays, immunostaining with lymph endothelium-specific marker D2–40 was not used in the present study because the staining was not routinely used for pathologic evaluation at the start of this trial. The other limitation of the present study was the difficulty in confirming true drug compliance. We checked drug compliance from the treatment diary every one to two months when the patient visited the hospital, but had no way of ensuring that the patient had made true declarations regarding drug intake. Investigators must keep in mind that all such oral administration studies conducted on an outpatient basis are subject to this problem in confirming true drug compliance.

## Conclusions

This prospective study has shown that 2-year oral tegafur-uracil is a feasible and promising treatment for LVI-positive stage IA NSCLC. In the current clinical guideline, adjuvant chemotherapy is not recommended for stage IA NSCLC less than 2 cm in size. In order to verify the true efficacy of this treatment, especially for LVI-positive stage IA tumor less than 2 cm in size, a randomized phase III study is recommended.

## Data Availability

The datasets used and/or analysed during the current study are available from the corresponding author on reasonable request.

## Abbreviations

LVI: Lymphovascular invasion
NSCLC: Non-small cell lung cancer
CEA: Carcinoembryonic antigen
DPD: Dihydropyrimidine dehydrogenase
5-FU: 5-fluorouracil
VEGF: Vascular endothelial growth factor
AST: Aspartate aminotransferase
ALT: Alanine aminotransferase
SAS: Safety analysis set
FAS: Full analysis set
